# Supplementary material for: Content-rich biological network constructed by mining PubMed abstracts
Source: BMC Bioinformatics. 2004 Oct 8;5:147. doi: 10.1186/1471-2105-5-147 (PMC528731; doi:10.1186/1471-2105-5-147)
Supplement: Additional File 2 — The original results of the above study (non-essential files are deleted to keep the file size under the limit set by BMC bioinformatics). [file 1471-2105-5-147-S2.bz2 › chilibotAdditionalFile2/dip40/331ID1328224E3927/html/TNFRSF1B_LTA.html]

 


 **TNFRSF1B** and **LTA** 
  
Found 38 abstracts in PubMed, retrieved 38.  
 

 What does Google say? 
 PDF only 
| .edu only 

---

**Interactive relationship** (e.g. stimulation, inhibition, etc)

**Neutral relationship**- Composed of 2 soluble TNF receptor p75 domains fused to human immunoglobulin, etanercept  [ **TNFRSF1B** ]  neutralizes the inflammatory cytokines TNF and lymphotoxin alpha  [ **LTA** ] .  Ref: 12463460 Clin Exp Rheumatol< MedlineTA>Clin Exp Rheumatol,
- Its receptors, TNF R55 and TNF R75  [ **TNFRSF1B** ] , bind in clefts formed by adjacent identical LT alpha  [ **LTA** ]  monomers.  Ref: 8878796 J Inflamm< MedlineTA>J Inflamm,
- LT consists of soluble LT alpha  [ **LTA** ]  3, which binds to TNF R1 and TNF R2  [ **TNFRSF1B** ] , and membrane LT alpha  [ **LTA** ]  1 beta 2, which binds to LT beta R.  Ref: 12882833 Int Immunol, 2003
- Cross linking of 125I TNF alpha and 125I TNF beta  [ **LTA** ]  to 293 TNF R2  [ **TNFRSF1B** ]  cells yielded predominant complexes with apparent molecular weights of 21 00 for TNF alpha and 20 00 and 24 00 for TNF beta  [ **LTA** ] , suggesting these complexes contain two or three TNF R2  [ **TNFRSF1B** ]  molecules.  Ref: 1328224 J Biol Chem, 1992
- TNF alpha and TNF beta  [ **LTA** ]  interact with TNF receptor TNF R, of which two subtypes have been described TNF R1 and TNF R2  [ **TNFRSF1B** ] .  Ref: 9174153 Tissue Antigens, 1997
- LT alpha  [ **LTA** ]  exists in its soluble form as a homotrimer, which like TNF only binds the TNF receptors, TNF R55 or TNF R75  [ **TNFRSF1B** ] .  Ref: 8920886 J Exp Med, 1996
- Tumor necrosis factor alpha TNF alpha and lymphotoxin alpha  [ **LTA** ]  LT alpha  [ **LTA** ]  induce pleiotropic cellular effects through low affinity 55 kDa type 1 receptors TNFR1, CD120a and high affinity 75 kDa type 2 receptors TNFR2  [ **TNFRSF1B** ] , CD120b  [ **TNFRSF1B** ] .  Ref: 9143243 J Neuroimmunol, 1997
- Scatchard analysis of 125I TNF alpha and 125I TNF beta  [ **LTA** ]  binding to sTNF R2 gave dissociation constants Kd of 0.3 and 0.75 nM, respectively, comparable to those observed for intact cell surface TNF R2  [ **TNFRSF1B** ] .  Ref: 8384489 Biochemistry, 1993
- TNF alpha and LT alpha  [ **LTA** ]  are both members of the TNF family of molecules and cause cell death by apoptosis following interaction with their counter receptors, the TNFR1 and TNFR2  [ **TNFRSF1B** ] , leading to a cascade of proteolytic events culminating in the blebbing of the cytoplasmic membrane, nuclear condensation and DNA fragmentation.  Ref: 11140465 Arch Immunol Ther Exp Warsz , 2000
- Four members of the tumor necrosis factor TNF ligand family, TNF alpha, LT alpha  [ **LTA** ] , LT beta, and LIGHT, interact with four receptors of the TNF nerve growth factor family, the p55 TNF receptor CD120a, the p75 TNF receptor CD120b  [ **TNFRSF1B** ] , the lymphotoxin beta receptor LT beta R, and herpes virus entry mediator HVEM to control a wide range of innate and adaptive immune response functions.  Ref: 10358762 Annu Rev Immunol, 1999
- Etanercept  [ **TNFRSF1B** ]  inhibits the activity of tumor necrosis factor and lymphotoxin alpha  [ **LTA** ] .  Ref: 11302411 Ann Pharmacother, 2001
- Lymphotoxin TNF beta  [ **LTA** ]  was also shown to stimulate human thymocyte proliferation, demonstrating that TNF beta  [ **LTA** ]  can initiate a biologic response that is mediated by TNF R2  [ **TNFRSF1B** ] .  Ref: 8409424 J Immunol, 1993
- In vitro, constitutive and stimulated production of TNF beta  [ **LTA** ] , IL 1beta, MIP 1beta, and IL 8 by normal mononuclear cells declined with etanercept  [ **TNFRSF1B** ] , detectable TNF alpha increased.  Ref: 11458980 Res Commun Mol Pathol Pharmacol, 2001

**Non-interactive relationship** (e.g. studied together, co-existance, homology, etc.)

- Our results indicate that LT alpha  [ **LTA** ]  induced inflammation is dependent on the interaction of LT alpha3 with TNFR1, and there is no obvious role for TNFR2  [ **TNFRSF1B** ] , since in its absence, LT alpha  [ **LTA** ]  induced inflammation is quantitatively and qualitatively similar to that seen in the wild type.  Ref: 9552007 J Immunol, 1998
- TNF, LT alpha  [ **LTA** ]  and LT beta and their receptors CD120a, CD120b  [ **TNFRSF1B** ] , TNFR RP interact in a complex fashion.  Ref: 8836404 Ann Oncol, 1996
- METHODS Immunohistochemical IHC staining was performed on formalin fixed, paraffin embedded tissues from 39 bronchogenic adenocarcinomas and 32 squamous cell carcinomas using polyclonal antibodies against TNF alpha, TNF beta  [ **LTA** ] , TNF R1, and TNF R2  [ **TNFRSF1B** ]  proteins.  Ref: 9669810 Cancer, 1998
- Spleen morphology and antibody response were investigated in wild type, TNFR1, TNFR2  [ **TNFRSF1B** ]  and TNF LT alpha  [ **LTA** ]  mice immunized with SRBC.  Ref: 8642347 J Exp Med, 1996
- Spleen morphology and antibody responses were investigated in wild type, TNFR1, TNFR2  [ **TNFRSF1B** ] , and TNF LT alpha  [ **LTA** ]  mice immunized with SRBC.  Ref: 8913932 J Inflamm< MedlineTA>J Inflamm, 1996
- This report analyzes the gene expression of bcl xs, bcl xl, bax alpha, bax beta, fadd, fas, fas ligand fas L, ice, TNF alpha, TNF beta  [ **LTA** ] , TNFR1, TNFR2  [ **TNFRSF1B** ] , TRAF1, TRAF2, TRAF3, cIAP2, and tradd at the level of mRNA in the single Reed Sternberg cells and their variants.  Ref: 9516144 Blood, 1998
- Shedding of the extracellular domain of TNF R2  [ **TNFRSF1B** ]  was induced by 4 beta phorbol 12 myristate 13 acetate but not by TNF alpha or TNF beta  [ **LTA** ] .  Ref: 1328224 J Biol Chem, 1992
- RESULTS Significant coexpression of TNF alpha, TNF beta  [ **LTA** ] , TNF R1, and TNF R2  [ **TNFRSF1B** ]  was observed in NSCLC significance range, P <.  Ref: 9669810 Cancer, 1998
- This activity of LT depends on membrane LT alpha  [ **LTA** ]  1 beta 2 and not soluble LT alpha  [ **LTA** ]  3, since DA virus persisted and induced inflammatory demyelination in LT beta R, but not TNF R1 or TNF R2  [ **TNFRSF1B** ] , mice.  Ref: 12882833 Int Immunol, 2003
- In this review we summarize the essential findings about the function of tumour necrosis factor TNF and its cognate receptors TNFR1 and TNFR2  [ **TNFRSF1B** ] , and lymphotoxin alpha  [ **LTA** ]  LT alpha  [ **LTA** ]  ligands in immune mediated CNS inflammation and demyelination.  Ref: 11004118 Brain, 2000
- CONCLUSIONS Although NSCLC exhibits strong coexpression of TNF alpha, TNF beta  [ **LTA** ] , TNF R1, and TNF R2  [ **TNFRSF1B** ] , there is a loss down regulation of TNF receptors in high stage tumors.  Ref: 9669810 Cancer, 1998
- Use of gene knockout mice indicates that depletion of BM B cells is dependent on TNF alpha, lymphotoxin alpha  [ **LTA** ] , and both TNF receptors, TNFR1 p55 and TNFR2  [ **TNFRSF1B** ]  p75.  Ref: 12444124 J Immunol, 2002
- There were significant differences between HAM patients and normal controls in the distributions of TNF promoter region polymophism at position 857, the LT alpha  [ **LTA** ]  gene NcoI polymorphism, and the T G substitution in exon 6 of the TNFR 2  [ **TNFRSF1B** ]  gene.  Ref: 11163081 Hum Immunol, 2000
- IL 2 induced tumor necrosis factor TNF beta  [ **LTA** ]  expression further analysis in the IL 2 knockout model, and comparison with TNF alpha, lymphotoxin beta, TNFR1 and TNFR2  [ **TNFRSF1B** ]  modulation.  Ref: 11157847 Int Immunol, 2001
- We studied tumor necrosis factor TNF, lymphotoxin alpha  [ **LTA** ]  LT alpha  [ **LTA** ] , and TNF receptors type 1 TNFR 1 and type 2 TNFR 2  [ **TNFRSF1B** ]  gene polymorphisms as well as HLA class II DRB1 alleles in Japanese patients with human T cell lymphotropic virus type I HTLV I associated myelopathy HAM n = 51, patients with adult T cell leukemia lymphoma ATL n = 48, asymptomatic HTLV I carriers n = 50, and HTLV I seronegative, normal controls n = 112.  Ref: 11163081 Hum Immunol, 2000
- In the present study, we examined 12 biallelic polymorphisms in the genes for tumor necrosis factor TNF alpha 488 G A, 238 G A, 308 G A, lymphotoxin LT alpha  [ **LTA** ]  720 C A, 365 C G, and 249 A G, determining haplotypes LT alpha1 to LT alpha4, tumor necrosis factor receptor 2  [ **TNFRSF1B** ]  TNF RII gb M32315 676 T G, 1663 A G, 1668 T G, 1690 C T, and interleukin IL 6 promoter 174 G C, intron 4 A G.  Ref: 11371414 Am J Respir Crit Care Med, 2001
- TNF and LT alpha  [ **LTA** ]  CD120a and CD120b  [ **TNFRSF1B** ] , CD30 CD30L, and CD40 CD40L are clearly critical elements in the deregulated network of interactive signals between H RS cells and surrounding bystander cells with membrane associated and cytokine mediated events.  Ref: 8836404 Ann Oncol, 1996
- We investigated the effect of a TNF and LT alpha  [ **LTA** ]  double deficiency on sleep in mice Ligand KO and compared their sleep with that of R1KO, TNFR2  [ **TNFRSF1B** ]  knockout R2KO mice, and wild type WT controls.  Ref: 12163535 J Neurophysiol, 2002
- To determine their role in vivo in lipopolysaccharide LPS and TNF induced skin inflammatory necrosis, TNFR1, TNFR2  [ **TNFRSF1B** ] , and TNF lymphotoxin alpha  [ **LTA** ]  LT alpha  [ **LTA** ]  deficient mice were used.  Ref: 9144075 J Inflamm< MedlineTA>J Inflamm, 2002
- The results suggest that the TNF LT alpha  [ **LTA** ]  gene region within the HLA class III of chromosome 6 and the TNFR 2  [ **TNFRSF1B** ]  gene region located on chromosome 1p36 might contribute to susceptibility to HAM,.  Ref: 11163081 Hum Immunol, 2000
- Antibody directed against Fas, TNFR60, TNFR80  [ **TNFRSF1B** ] , LTBR, and LT alpha  [ **LTA** ]  failed to inhibit receptor induced death.  Ref: 9021927 J Leukoc Biol, 1997
